# Geographical Analysis for Detecting High-Risk Areas for Bovine/Human Rabies Transmitted by the Common Hematophagous Bat in the Amazon Region, Brazil

**DOI:** 10.1371/journal.pone.0157332

**Published:** 2016-07-07

**Authors:** Fernanda A. G. de Andrade, Murilo N. Gomes, Wilson Uieda, Alberto L. Begot, Ofir de S. Ramos, Marcus E. B. Fernandes

**Affiliations:** 1 Campus de Pesquisa, Museu Paraense Emílio Goeldi, Belém, Pará, Brazil; 2 Escritório de Defesa Agropecuária de São Paulo, Coordenadoria de Defesa Agropecuária, São Paulo, Brazil; 3 Departamento de Zoologia, Instituto de Biociências, Universidade Estadual Paulista “Júlio de Mesquita Filho”, Botucatu, São Paulo, Brazil; 4 Departamento de Endemias, Secretaria Executiva de Saúde Pública do Estado do Pará, Pará, Brazil; 5 Laboratório de Virologia, Laboratório Nacional Agropecuário do Pará, Pará, Brazil; 6 Laboratório de Ecologia de Manguezal, Universidade Federal do Pará, Campus de Bragança, Pará, Brazil; Linneaus University, SWEDEN

## Abstract

**Background:**

The common hematophagous bat, *Desmodus rotundus*, is one of the main wild reservoirs of rabies virus in several regions in Latin America. New production practices and changed land use have provided environmental features that have been very favorable for *D*. *rotundus* bat populations, making this species the main transmitter of rabies in the cycle that involves humans and herbivores. In the Amazon region, these features include a mosaic of environmental, social, and economic components, which together creates areas with different levels of risk for human and bovine infections, as presented in this work in the eastern Brazilian Amazon.

**Methodology:**

We geo-referenced a total of 175 cases of rabies, of which 88% occurred in bovines and 12% in humans, respectively, and related these cases to a number of different geographical and biological variables. The spatial distribution was analyzed using the Kernel function, while the association with independent variables was assessed using a multi-criterion Analytical Hierarchy Process (AHP) technique.

**Findings:**

The spatiotemporal analysis of the occurrence of rabies in bovines and humans found reduction in the number of cases in the eastern state of Pará, where no more cases were recorded in humans, whereas high infection rates were recorded in bovines in the northeastern part of the state, and low rates in the southeast. The areas of highest risk for bovine rabies are found in the proximity of rivers and highways. In the case of human rabies, the highest concentration of high-risk areas was found where the highway network coincides with high densities of rural and indigenous populations.

**Conclusion:**

The high-risk areas for human and bovine rabies are patchily distributed, and related to extensive deforested areas, large herds of cattle, and the presence of highways. These findings provide an important database for the generation of epidemiological models that could support the development of effective prevention measures and controls.

## Introduction

All three species of hematophagous bats of the family Phyllostomidae, subfamily Desmodontinae, are found in Latin America, while only the common hematophagous bat–*Desmodus rotundus* (E. Geoffroy Saint-Hilarie, 1810)–feeds preferentially on the blood of mammals, although all the hematophagous species have been diagnosed positive for rabies in South America [1]. The specialization for blood-feeding in this species is regarded as a high-level trophic adaptation, which is reflected in the relatively low densities of this species in most natural environments [2,3]

*Desmodus rotundus* is nevertheless becoming relatively common in regions with large herds of domestic cattle [4, 5], and some authors have referred to a strict ecological relationship between the populations of these two types of mammals [3,6,7]. Holmes *et al*. [8] concluded that *D*. *rotundus* is favored by the presence of an abundant and easily-exploited feeding resource represented by the cattle, which may result in an increase in survival rates and population density, especially in areas where appropriate roosts are available [9].

*Desmodus rotundus* is an important vector of rabies, which is a fatal disease for all mammals, including the bat itself, with contamination resulting from the contact between individuals (including those of other bat species) in the different roosts typically utilized by the animals within their home ranges [10,11]. In natural areas, the intra- and interspecific contamination among bats seems to occur more likely due to the influence of the biological characteristics of these mammals (immunology, physiology, and genetics) as well as local environmental conditions, maintaining the virus exclusively within populations of wild animals [6].

In Pavlovsky’s [12] theory of “natural outbreaks”, the influence of human activities on natural environments results in local outbreaks, which are limited to a single type of environment or biogeographic landscape, due to the correlations among the different variables involved in the process. In the specific case of rabies transmitted by *D*. *rotundus*, however, Gomes [13] concluded that the areas impacted by agricultural activities might produce new features or variables, which create the conditions for the establishment of areas with different degrees of risk for bovine rabies.

In Brazil, the transmission of rabies from blood-eating bats to humans is more common in rural areas. The records from the Brazilian state of Pará are concentrated in 2004 and 2005, and include riverside communities from Marajó Island and coastal areas of the northeastern portion of the state [14, 15]. In the Amazon basin, different rural areas vary considerably in their relationship with the disease and its epidemiology, with the residents of some areas reporting frequent bites, and considering this to be a normal part of daily life [9]. The concept of rabies in the region’s traditional communities is also an important question. Finally, the lack of doors and windows in many rural residences facilitates access for the bats [16].

In the Amazon region, a number of variables may contribute to the maintenance of the rabies virus [9], including both the availability of food and roosts for *D*. *rotundus*. The abundance of potential roosts (culverts, bridges, abandoned mines, buildings, and ovens for coal production) may increase as a result of land use patterns and represents a type of transformation of the environment that may invoke risks for human health [17].

In addition, it is important to note that rabies is a lethal disease that generates enormous financial expenditures due to the costs of control measures and the vaccination and treatment of human victims. These costs also include the control of this zoonosis in herbivores, which reach millions of dollars in Latin America [18], in addition to the lowered productivity that include a reduction in the output of milk, and the quality of the leather, as well as an increase in the risk of secondary infections [19].

The analysis of rabies cases requires the consideration of different types of information, including regional and demographic parameters, as well as land use patterns and their socio-environmental impacts. The study of the dispersal of cases in relation to the distribution of areas of risk relies on analytical tools that can integrate the spatial and temporal aspects of these variables systematically [20]. In the Amazon, these tools must also provide a basis for the evaluation of potential associations with natural or anthropogenic modifications of the environment [21].

Over the past decade, information technology has been widely used for the evaluation of the incidence and distribution of rabies [22–28]. In particular, Geographic Information Systems (GIS) have provided a highly efficient environment for the management, storing, analysis, and mapping of data from the public health sector [29]. Given all these considerations, the present study evaluates the variation in the distribution and density of cases of rabies in humans and bovines in the eastern Amazon basin between 1999 and 2008. The data were also used to define risk areas for the transmission of the virus by *D*. *rotundus* within the study area. The main objective was to determine the spatiotemporal distribution and high-risk areas for bovine/human rabies in order to provide a solid scientific foundation for the development of effective strategies of monitoring and control of this zoonosis, and contribute to the mitigation of its socioeconomic and environmental impacts.

## Material and Methods

### Rabies database

**Fig 1:**
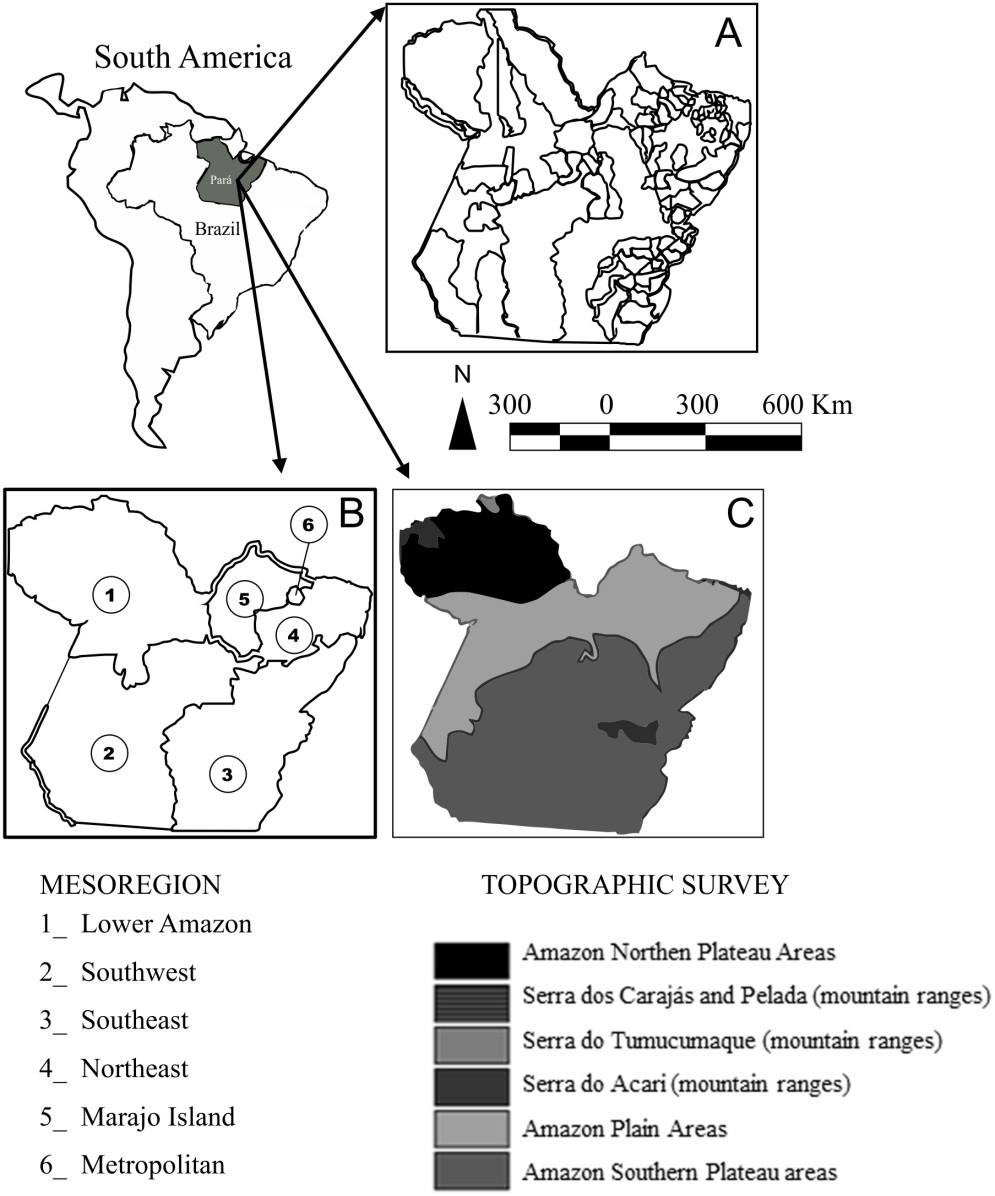
Map of the state of Pará, Brazilian Amazon. Political, economic, and geographical division of the state of Pará represented by: (A) municipalities, (B) mesoregions, and (C) main topographic features [30]. Scale 1:250,000.

The dataset of the cases of rabies transmitted by the common hematophagous bat to humans and bovines used in the present study was gathered from the Endemisms Department of the Executive Secretariat for Public Health in the state of Pará (SESPA) and the Pará State Agricultural Defense Agency (ADEPARA), respectively, which are responsible for the information system of infectious diseases of mandatory notification in the State of Pará, Brazilian Amazon (Fig 1).

Each recorded death caused by bat-transmitted rabies was geo-referenced by municipality. In this case, history of cases in humans and bovines was compiled for each municipality between 1999 and 2008. This period was selected due to the occurrence of the highest rates of rabies cases transmitted by *D*. *rotundus* yet recorded in Brazil.

The rabies cases were reported in 18 municipalities representing four different mesoregions, and included 49 confirmed cases in humans and 154 in bovines (Table 1). In 38 of the human cases, the rabies resulted in the death of the patient, of which 21 were attributed to attacks by *D*. *rotundus*. In addition, the under-reporting of cases may be a potential bias for the analysis of this zoonosis in Brazilian Amazonia, reinforced by the practices adopted by the local public authorities for the control of outbreaks. Specifically, where deaths were reported, programs of vaccination (humans and cattle) and control measures of the local *D*. *rotundus* population are implemented to control the dispersal of the virus.

**Table 1:** Municipalities of the Brazilian state of Pará (and their respective mesoregion) in which positive cases of human and bovine rabies were recorded between 1999 and 2008.

Municipality	Mesoregion	Number of positive cases in:	
		Humans	Bovines
**Augusto Corrêa**	Northeast	15	14
**Bragança**	Northeast	0	21
**Capitão Poço**	Northeast	0	10
**Conceição do Araguaia**	Southeast	0	17
**Dom Eliseu**	Southeast	1	0
**Floresta do Araguaia**	Southeast	1	0
**Irituia**	Northeast	2	0
**Itupiranga**	Southeast	1	0
**Mãe do rio**	Northeast	0	8
**Marabá**	Southeast	2	0
**Medicilândia**	Southeast	0	8
**Portel**	Marajó	15	0
**Redenção**	Southeast	1	17
**Rondon do Pará**	Southeast	1	0
**São João do Araguaia**	Southeast	1	0
**Viseu**	Northeast	9	15
**Tracuateua**	Northeast	0	37
**Ulianópolis**	Southeast	0	7
**TOTAL**		49	154

The variables related to the cases of rabies in both humans and bovines were organized in two groups: (Group A) i—municipality, ii—year of the record, iii—organism infected (human/bovine), and iv—source of the infection, and (Group B) i—ecological variables and ii—biological variables. Only two independent variables were considered for the analysis of the bovine cases: (i) deforestation between 1998 and 2008 [31] and (ii) the size of the bovine herd [32]. In the case of human infections, the variables analyzed were (i) the number of rural [33] and indigenous [34] populations and (ii) the number of rural [35] and indigenous localities [36]. As variables, the drainage basins [37], and municipal highway networks [38] generated features shared by these two categories.

To evaluate the intensity of the relationship between the recorded cases and the variables presented above, three analyses were conducted, for which the geo-referenced municipality data had to be aggregated mathematically.

### Analysis of rabies density by the Kernel Quartic Function

The records of rabies cases in humans and bovines in Pará during the period between 1999 and 2008 were geo-referenced and the number of cases were linked by the municipality polygons [39]. Of the total number of cases in humans (n = 38), 21 infections (55%, 95% CI: 37.85–72.67%) were recorded in Portel, Viseu, and Augusto Corrêa in 2004 and 2005, and attributed to *D*. *rotundus* bites. However, only 16 cases (Viseu, n = 1 [40], Portel, n = 5 [16], and Augusto Corrêa, n = 10) were confirmed by antigen analysis, indicating the presence of the VAg3 variant of the virus, which is common in this bat species [41].

The magnitude of the number of deaths from rabies infection, over ten years of record, was presented in five thematic maps, after conjugation of every two years, and interpolated in the TerraView 4.2.2 program [42] using the Kernel function on a digital map of the state of Pará provided by GISMAP [43]. The objective of this approach was to map the dispersal of rabies within the state in the context of the different categories analyzed. The Kernel function is a nonparametric technique, which provides a systematic approach for the interpretation of a process and the resulting clusters of events.

### Identification of areas of risk for rabies

The variables applied for the evaluation of the distribution of cases of rabies in humans and bovines were transformed when needed (Table 2). The thematic maps used for the Analytical Hierarchy Process (AHP) technique in humans and bovines represented the independent variables considered for this type of analysis. These maps were classified according to their relevance level—Low, Medium I, Medium II, and High—providing relative weights for each theme (Table 2). The consistency of these weights was represented by an index, which varied from 0 (absolute consistency) to 1 (no consistency). In general, the weighting was relatively consistent (in humans = 0.064, in bovines = 0.072), providing a reliable basis for the calculation of the classes for each variable, with values ranging from 0 to 1 (Table 3). Each of these operational stages was run in SPRING 5.1.5 [44].

**Table 2:** Variables associated with rabies in humans and bovines, and the weighting attributed to each variable for analysis.

Category	Variable	Description of variable	Application method	Source access	Assigned weight
Humans	Road buffer	Distance of the area from main highways	Geographic location	[38]	0.08
	Buffer of hydrographic conditions	Distance of the area from the main rivers	Geographic location	[37]	0.08
	Geographic distribution of rural human populations and indigenous	Total population	Interpolation of a data using weighted average	[33] [36]	0.24
	Density of rural and indigenous settlements	Total number of farms and indigenous communities	Kernel function	[34] [35]	0.55
Bovines	Road buffer	Distance of the area from main highways	Geographic location	[38]	0.06
	Buffer of hydrographic conditions	Distance of the area from the principal rivers	Geographic location	[37]	0.06
	Livestock distribution	Total number of cattle	Interpolation of data using Nearest Neighbor analysis	[32]	0.26
	Deforestation	Proportion of deforested area	Geographic location	[31]	0.57

**Table 3:** Weighting defined for the variables analyzed in both bovines and humans.

Variable	Description of variable	Features	Assigned weighting
**Roads/ hydrographic conditions**	Distance from areas of recent outbreaks to highways/rivers	> 100 km	0.5
		60 to 100	0.5
		30 to 60	0
		0 to 30	0
**Geographic distribution of rural human populations and indigenous settlements**	Total number of rural human populations and indigenous settlements	Low	0
		Medium I	0.5
		Medium II	0.8
		High	1
**Livestock distribution**	Total number of cattle	77778 to 582416000	0.1
		582416000 to 1530576000	0.3
		1530576000 to 3900761000	0.5
		3900761000 to 12597414000	0.8
**Deforestation**	Area deforested	143 km^2^	1

## Results

### Annual spatial distribution and density of rabies cases in humans and bovines

**Fig 2:**
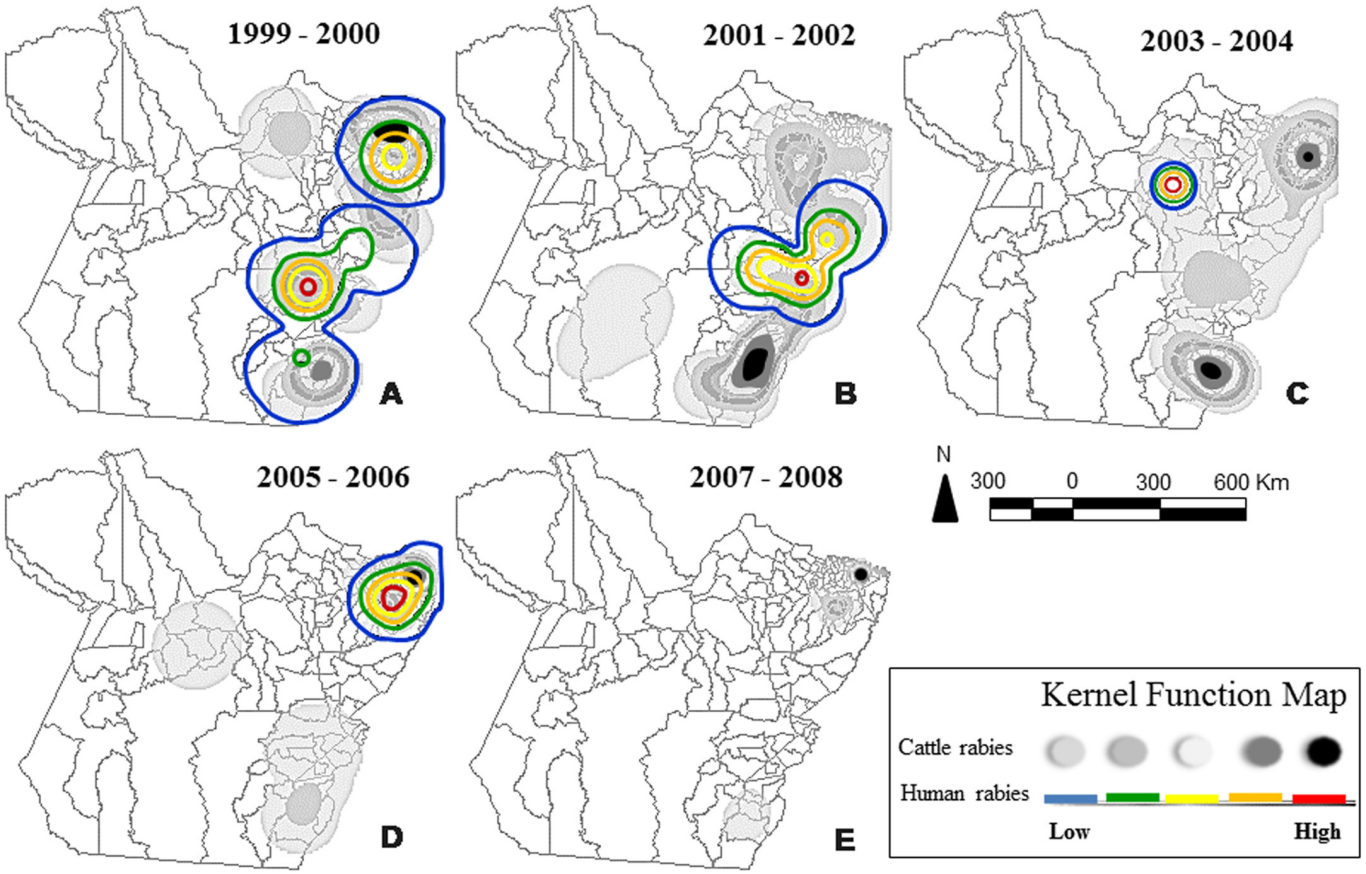
Map of the bat-transmitted rabies in the Brazilian Amazon. Spatial distribution of bovine/human rabies in two-year intervals between 1999 and 2008 in the state of Pará, Brazil.

Between 1999 and 2008, the cases of rabies transmitted by *D*. *rotundus* were distributed primarily in eastern Pará. During the subsequent period, there was a convergence of the hot (high frequency of cases) zones for both humans and bovines (Fig 2). In humans, the initial records referred to transmission by dogs and cats from 1999 to 2002 (Fig 2A and 2B), which are replaced by major outbreaks in Latin America caused by bats [16].

The number of deaths in cattle caused by the rabies virus in Pará tended to decline after 2005 (Fig 2D), and the hot zones of human deaths disappeared after 2007 (Fig 2E). Both these trends were the result of the efforts of the local public health authorities, which were essential for the reduction of the bovine hot zone in southeast mesoregion, and subsequently, the reduction of cases in the northeastern mesoregion. In humans, the control of outbreaks was effective initially in the southeast, followed by the Marajó mesoregion, culminating in the elimination of the northeastern hot zone (Fig 2E).

### Identification of the risk areas for human and bovine rabies

**Fig 3:**
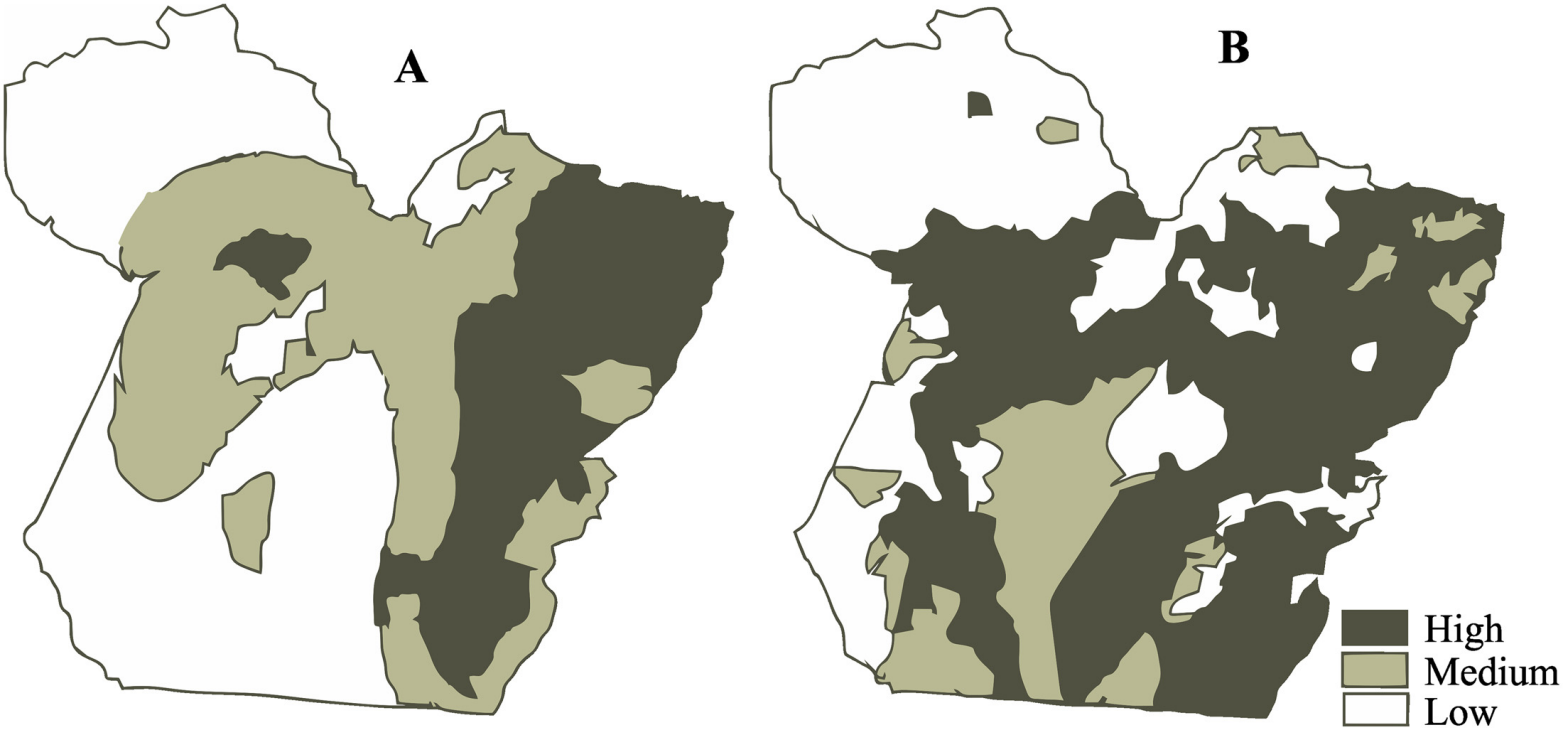
Map of the risk areas for rabies in the Brazilian Amazon. Spatial distribution of risk areas of bat-transmitted rabies in the state of Pará, Brazil: (A) bovine and (B) human.

Based on the variables analyzed in the present study, the areas with the highest risk for the transmission of *Lyssavirus* by *D*. *rotundus* are concentrated in extensive deforested areas, as well as those with the large herds of cattle and the largest numbers of highways. The areas of highest risk for bovine rabies are patchy, but less widely dispersed, and in the west of the state, they are invariably found in the proximity of major rivers and highways (Fig 3). However, the medium risk areas are grouped in the center of the state, while the low risk areas are concentrated along the borders with the neighboring states of Amazonas and Amapá. It is important to note here that the spatial distribution of bovine rabies together with that of the associated variables indicate the importance of the combination of variables for the determination of risk levels, especially in the southeastern mesoregion.

In the case of human rabies, the highest concentration of high-risk areas was found in the eastern portion of Pará, where the highway network and high densities of rural and indigenous populations coincide. The medium risk areas coincide with much of the northwestern portion of the state, which is dominated by the basins of major rivers, such as the Amazon, Araguaia and the Tocantins. However, this area does not extend to the southwestern portion of the state, which is characterized by low risks of infection with rabies for humans.

## Discussion

The present study used spatial statistical analyses to interpret the spatiotemporal progression of the outbreaks of human and bovine rabies that occurred in the Brazilian Amazon region between 1999 and 2008. The Kernel function indicated a high concentration of cases in eastern Pará, related to different environmental variables. The AHP technique identified zones of intensity, with a high potential for the occurrence of rabies outbreaks in the region. We then described the progression of rabies outbreaks over the years and concluded that the diversity, dynamics, and variation in the use of environment in the Brazilian Amazon are constraints for the development of a more widely applicable model for the prediction of the transmission of rabies by *D*. *rotundus*.

The historical data series (1999–2008) analyzed in the present study confirmed the highest number of cases of human rabies for the Brazilian Amazon region, as well as an intimate relationship between the density of positive cases and the expansion of cattle herds in the eastern portion of the state of Pará. Cattle ranching in this state has increased considerably since 1991, and in the Brazilian Amazon region as a whole, and the production of beef is now second only to that of Mato Grosso [45]. The state’s southeastern and southwestern mesoregions are the principal producers, accounting for 67% and 16%, respectively, of the state’s output [46]. In addition, the number of recorded cases in bovines decreased progressively over the years, probably as a result of the efforts to control the spread of the disease through public health campaigns, including vaccination programs and the control of *D*. *rotundus* populations, in particular following a number of deaths in human patients in specific local areas in 2004 and 2005.

The records of bat-borne rabies in humans in the Brazilian Amazon region constitute the highest number of cases of any region in the country, or anywhere in Latin America, distributed primarily between the Marajó (Portel) and Northeast (Viseu and Augusto Corrêa) mesoregions, which are 530 km apart. The traditional populations of the Amazon basin have more irregular distribution of residences, which are often found in isolated locations [47], generally in the vicinity of aquatic environments, that are considered to be an important source of food resources [48]. The houses typically lack doors and windows and are surrounded by livestock and fruit trees; furthermore, there is a lack of adequate knowledge for the prevention of infection in this region [16]. Bat bites are considered as a normal feature of daily life in most traditional communities, and as long as there is no risk of infection with rabies, they can be considered to be part of the interaction of the population with the natural environment.

The Lower Amazon and Southeast mesoregions of the state of Pará cover vast areas that contain many herds of cattle [46], and are characterized by low deforestation rates, but also reduced economic indices during the study period, between 1999 and 2008 [30]. During this period, the economies of these mesoregions were supported primarily by fisheries and mining, respectively [47], although cattle ranching was expanding rapidly, especially on the lower Amazon [46].

In addition to the different types of livestock, the relationship between Amazonian landowners and both the physical environment and the disease also plays an important role in the epidemiology of *Lyssavirus* [48]. The residents of rural areas are fundamental to effective public health monitoring and education by the relevant authorities [49]. However, the technical manual published by MAPA [50] states that, in some cases, the persistent occurrence of rabies in bovines may be due to the “unsatisfactory performance of the state program for the control of rabies in herbivores”. This may be a result from the under-reporting of cases related to (i) the rudimentary facilities for clinical diagnosis in the field, and (ii) the lack of any systematic collection of samples for laboratory analyses [49].

Even so, the ample geographic distribution of *D*. *rotundus* and the free circulation of the virus among bats are two of the factors that contribute most to the persistence of the disease in the Brazilian Amazon region [51, 52]. The persistence of outbreaks in certain areas is also related directly to the performance of the local public health authorities responsible for the control of the disease. In practice, the control of this zoonosis is dependent on the identification of the so-called sentinel events, that is, the first death, which normally occur in a bovine, after which the entity responsible for the control of rabies in humans is involved. Ideally, however, an effective preventive strategy would be based on the monitoring and control of the occurrence of attacks by hematophagous bats in both humans and cattle, principally in the high risk zones, given that any of the two factors on their own are insufficient to actually cause rabies outbreaks, but rather to the interaction between bat attacks and associated variables.

The major areas of deforestation in Pará are concentrated in the northeastern portion of the state [47], mainly in the coastal zone that is a mosaic of mangroves forests adjacent to anthropogenic landscapes, where cattle ranches represent only 8.6% of the state of Pará [32]. Gomes *et al*. [53] demonstrated that the presence of pastures in close proximity to forest remnants favored the access of bats to the cattle. Mangroves are considered to be an important environment for many mammalian species, including bats [54]. In the Amazon region, *D*. *rotundus* has been captured during the night in mangrove forest in Pará [55], where tidal creeks and other bodies of water may provide access corridors linking different habitats. This may favor the dispersal of blood-feeding bats between populations [56], as well as reinforcing their adaptability to changes in habitat characteristics [57].

The use of Warfarin to control *D*. *rotundus* populations may cause—among other effects—a reduction in the number of females and the consequent dispersal of the males to new roosts [58]. Given that the spread of rabies outbreaks may be favored by the interchange of bats between colonies [59], populations with infected individuals that are easily accessible may promote outbreaks of the disease more efficiently and over a shorter time scale. It is also important to note that, as found in previous studies, infection rates in a population may be related to specific characteristics, such as genetic diversity, the physiological condition of individuals [60,61], and immunity levels [6,62]. In this case, some *D*. *rotundus* populations may contain a relatively large proportion of individuals susceptible to infection with rabies due to intense behavioral stress and modifications to the landscape.

The AHP analysis of environmental and socioeconomic variables for the state of Pará revealed a dynamic epidemiological mosaic in terms of the distribution of risk areas. The presence of high-risk zones in areas with few reported cases indicates that these areas may be vulnerable to outbreaks. Even in the areas with relatively small herds of cattle, however, such as the northeastern mesoregion, the traditional lifestyle of the rural population, combined with impacts on the environment, provides a potential scenario for eventual outbreaks in humans.

In the southwest of the state, areas of medium risk for bovines are gradually shifting to high risk areas, with the exception of a large central zone that coincides exactly with indigenous reservations and environmental protection areas, as shown on an indigenous land map elaborated by the National Indian Foundation (FUNAI) [63]. The evidence indicates a low number of rabies cases overall, and especially in southwestern Pará, possibly because the large herds of cattle found in this region are concentrated in a small number of very large ranches, where public health authorities and the landowners themselves conduct more effective monitoring and control of zoonoses.

The relatively large number of human cases recorded in 2004 in the Marajó mesoregion, which is characterized by low population densities and widely-dispersed rural properties and indigenous reservations, coincided with the first significant reduction in the region’s bovine herds. Following a period of stability from 1999 onwards, the local herds decreased in size by approximately 10% by 2004 [48], which may have forced *D*. *rotundus* to seeking out new feeding resources, including humans. Given the number of cases of human and bovine rabies recorded in the Marajó and Northeast mesoregions of the state, a number of measures will be necessary in order to avoid new outbreaks, including: (i) reinforced monitoring of attacks on humans and animals, (ii) more systematic recording of cases, (iii) vaccination of affected individuals, (iv) surveys of local bat populations, and (iv) effective control of *D*. *rotundus* populations. The collection of biological samples (brain, brown fat, and blood) from bat specimens for the immunological and molecular monitoring of this host would also be recommended.

The results of the present study cover only some of the predictive variables of the spatial-temporal distribution of the rabies virus transmitted by hematophagous bats during a specific period. Given this, they provide guidelines for the development of a more effective and widely applicable model for the prediction of the transmission of rabies by *D*. *rotundus*. In any case, it is important to include data on the socio-historical and biological characteristics of each region for a more systematic and effective analysis of parameters, which can be applied to any region in the Brazilian Amazon or elsewhere in Latin America. Our results are also consistent with the suggestions of Stoner-Duncan [64] to address the problem of vampire bat–transmitted rabies to humans and domestic animals in Amazonia, with the assessment of the potential impact of ecological changes and human interventions in the zoonotic disease transmission.

Ultimately, a model for the prediction of the risk of occurrence of rabies in humans or bovines depends on diversity and dynamics of the environment, and the land use practices found in different regions. In a study of malaria, it was found that the definition of a landscape or macro-landscape of the occurrence of outbreaks, and thus risks, will be associated with local changes over time [17].
